# Identification and analysis of pig chimeric mRNAs using RNA sequencing data

**DOI:** 10.1186/1471-2164-13-429

**Published:** 2012-08-28

**Authors:** Lei Ma, Shulin Yang, Weiming Zhao, Zhonglin Tang, Tingting Zhang, Kui Li

**Affiliations:** 1 The Key Laboratory for Domestic Animal Genetic Resources and Breeding of Ministry of Agriculture of China, Institute of Animal Science, Chinese Academy of Agricultural Sciences, Beijing, 100193, P. R. China; 2 College of Life Science, Shihezi University, Xinjiang, 832000, P. R. China

**Keywords:** Chimeric mRNA, Trans-splicing, RNA-sequencing, CTCF, Pig

## Abstract

**Background:**

Gene fusion is ubiquitous over the course of evolution. It is expected to increase the diversity and complexity of transcriptomes and proteomes through chimeric sequence segments or altered regulation. However, chimeric mRNAs in pigs remain unclear. Here we identified some chimeric mRNAs in pigs and analyzed the expression of them across individuals and breeds using RNA-sequencing data.

**Results:**

The present study identified 669 putative chimeric mRNAs in pigs, of which 251 chimeric candidates were detected in a set of RNA-sequencing data. The 618 candidates had clear trans-splicing sites, 537 of which obeyed the canonical GU-AG splice rule. Only two putative pig chimera variants whose fusion junction was overlapped with that of a known human chimeric mRNA were found. A set of unique chimeric events were considered middle variances in the expression across individuals and breeds, and revealed non-significant variance between sexes. Furthermore, the genomic region of the 5^′^ partner gene shares a similar DNA sequence with that of the 3^′^ partner gene for 458 putative chimeric mRNAs. The 81 of those shared DNA sequences significantly matched the known DNA-binding motifs in the JASPAR CORE database. Four DNA motifs shared in parental genomic regions had significant similarity with known human CTCF binding sites.

**Conclusions:**

The present study provided detailed information on some pig chimeric mRNAs. We proposed a model that trans-acting factors, such as CTCF, induced the spatial organisation of parental genes to the same transcriptional factory so that parental genes were coordinatively transcribed to give birth to chimeric mRNAs.

## Background

Chimeric mRNAs fused by two previously separate genes located on different genomic loci may allow a limited number of genes to encode a substantially large number of mRNAs and proteins. They are expected to increase proteomic diversity through chimeric proteins or altered regulation. As a consequence, gene fusion can change the properties of precursor proteins and can even perturb normal regulatory pathways and initiate or stimulate neoplastic cell growth. A well-known example is the *BCR-ABL1* fusion gene, which is the result of the chromosomal translocation t(9; 22)(q34; q11) and is responsible for 90% of chronic myelogenous leukemia cases [1]. In this sense, chimeric genes can be used as desirable therapeutic targets for cancers. For instance, matinib mesylate (Gleevec, Novartis) can target the oncogenic kinase activity of *BCR-ABL1* in chronic myeloid leukemia [2-4]. Therefore, the identification and analysis of novel chimeric genes will pave the way for a greater understanding of the role of gene fusion.

Chromosomal translocation is generally responsible for the generation of some chimeric mRNAs in cancer cells. Therefore, chimeric mRNAs are often viewed as potential diagnostic biomarkers for tumours caused by chromosomal translocation. However, a low amount of a chimeric RNA (*JAZF1-JJAZ1*) was detected in normal endometrial tissues, joining the *JAZF1* gene on chromosome band 7p15 to the *JJAZ1/SUZ12* gene on chromosome band 17q21 [5]. Chimeric RNAs and proteins are identical to those produced from a chromosomal rearrangement found in human endometrial stromal tumours [5]. The explanation generally offered for this finding is that specific chromosomal rearrangements occur within small numbers of cells in healthy tissues. However, no rearranged bands t(7;17)(p15;q21) were detected in normal cells [5]. Given the absence of any detectable rearranged DNA in cells producing chimeric RNAs, the obvious explanation is the rearrangement at the RNA level. After incubation of mixed extracts from a human endometrial stromal cell line and from a rhesus monkey fibroblast cell line, rhesus *JAZF1* exons were joined to human *JJAZ1* exons, implying that the *JAZF1-JJAZ1* RNA is a result of trans-splicing [5].

In eukaryotes, trans-splicing is a special event in RNA processing where exons from two different primary RNA transcripts are joined from end to end and then ligated. In simulating the RNA cis-splicing mechanism, a cDNA is thought to be generated by trans-splicing when it is aligned to multiple non-contiguous genomic loci and the fusion junction obeys canonical GU-AG splice site. However, how precursor genes find each other before splicing remains to be elucidated, and where the trans-splicing event takes place is still poorly understood.

Some chimeras are derived from a non-spliceosome mechanism [6]. Short homologous sequences are proposed to be associated with the generation of chimeric mRNAs in eukaryotes, suggesting that the ‘misaligns’ of short homologous sequences could guide the chromosomal interaction for the proximity of distal genes [7]. In addition, read-through/splicing is another way of generating chimeric mRNAs [8-11]. In this process, an mRNA starts from the upstream gene, reads through intergenic regions, and ends at a termination point of the adjacent downstream gene, with the region in between removed by splicing. However, read-through/splicing cannot explain the chimeras derived from different chromosomes or opposite strands. Some chimeric mRNAs may have originated from the strand-switching feature of the reverse transcripatase [12]. In some cases, chimeric mRNAs are considered as artefacts from the reverse transcription polymerase chain reaction (RT-PCR) [12].

The presence of chimeric mRNAs in normal cells is a critical issue because the important pathways in normal cells would be disrupted by the potential therapy targeting chimeric mRNAs and proteins. The identification of chimeric mRNAs in normal cells will provide a wealth of biological information for this issue. The pig (*Sus scrofa*) is an economically important species and a potential medical model for some human health issues [13]. Therefore, research on chimeric mRNAs in normal cells can benefit from pigs. Results from the present study provide the first broad overview of chimeric mRNAs in pigs, and their analysis in normal tissue will aid in the further understanding of the molecular mechanisms of gene fusions.

## Results

### Identification of putative chimeric RNAs

**Figure 1:**
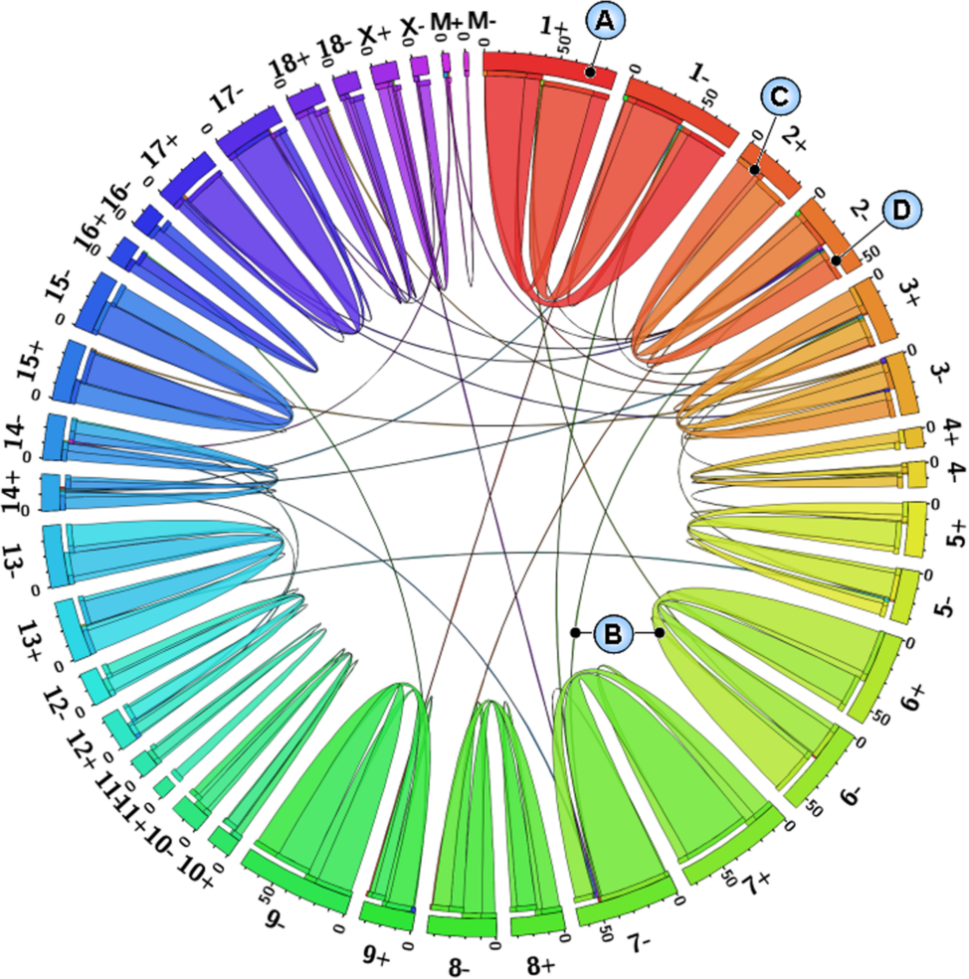
**Circular representation of the genome-wide distribution of putative chimeric mRNAs.** The outermost labels indicate the chromosome name with strand orientation. Each coloured segment (**A**) in the outermost circle encodes a chromosomal strand. Each bin in a segment represents ten events. Inner Ribbons (**B**) indicate the associated fusions from the 5^′^ partner to the 3^′^ partner. Ribbons are coloured according to the chromosomal strands in which the 5^′^ partners locate. Ribbons start from the 5^′^ partners with ribbon ends (**C**) coloured regarding the destination and stop at the 3^′^ partners with gaps (**D**) between the ribbons and associated segments. Ribbon size encodes the relative abundance of the associated fusion, that is, the count of putative chimeric mRNAs.

After inspecting the chromosomal loci of mRNAs from the pigs, many mRNAs were located on non-contiguous positions. An issue whether any of these mRNAs are real chimeras fused from two previously separate transcripts was raised. Highly qualitative alignments of mRNAs to the *S. scrofa* chromosomes (SGSC Sscrofa9.2/susScr2, Nov. 2009) in the Genome Browser database of the University of California Santa Cruz (UCSC) [14,15] may shed light on this issue. Alignments having at least 96% sequence identity and a minimum length of 100 nt were used in this study. We only used mRNAs that were matched on two non-contiguous loci to ensure that inferred chimeras were results of actual fused transcripts rather than alignment artefacts. Alignments from two non-contiguous loci were required not to possess long similar sequences at the putative junction sites to discard false positive results from homologous, paralogous, or random spurious hits. In this step, we only allowed overlaps or gaps of up to 10 nt within the fusion junction to accommodate small errors in alignment that occur at the edges of the alignment. Consequently, 669 mRNAs were inferred as putative chimeras (Additional file 1), including 27 inter-chromosomal and 642 intra-chromosomal junction events. In the intra-chromosomal events, 494 and 148 mRNAs were inter-strand and intra-strand junction events, respectively. Only three candidates involved mRNAs from the mitochondrial genome. Figure 1 displays the distribution of putative chimeric mRNAs in chromosomes, showing that inter-strand events are overrepresented in the set of predicted chimeric mRNAs.

For the confirmation of a hybrid transcript candidate, we inspected whether the fusion point corresponded to a pair of known splice sites. We separately extracted the chromosomal DNA sequences of the 5^′^ and 3^′^ partners of an inferred chimera and then connected the two non-contiguous genomic sequences to an artificially fused genomic sequence. Each inferred chimera was aligned to the corresponding artificially fused genomic sequence using the SIM4 program [16] to take into account consensus splice signals. The alignment around the fusion point was checked. Only the fusion points that were aligned precisely, without a gap or overlap, were retained. In addition, the reading frame must have structural integrity. Finally, 618 candidates had clear trans-splicing sites, 537 of which obeyed the GT/AG rule (Additional file 1).

To confirm further the trans-splicing events, 48 chimeric candidates were randomly selected for the RT-PCR assay using RNA from a number of tissues (see Methods). An RT-PCR product was required to span the fusion point. Through this assay, 36 out of randomly selected candidates showed identity with the expected fusion sequences (Additional file 2). Given that the transcription of mRNAs may vary in different tissues or stages of life, the selected samples for the RT-PCR assay may not be suitable for their expression. In addition, all mRNAs used in the present study have prior biological studies annotated in databases of the UCSC and the NCBI (National Centre for Biotechnology Information). Thus, the rate detected by the RT-PCR assay might underestimate the positive rate of chimeric mRNA identification. The use of expressed sequence tag (EST) and RNA-sequencing data from more tissues or stages would supply the gaps of the RT-PCR assay.

Putative chimeras were aligned to ESTs downloaded from the UCSC database to seek support from external experimental evidences and verify the putative fusion junctions. If at least 20 nt of the sequence on either side of a putative fusion point overlap with the ESTs, this candidate was retained for further analysis. The 431 candidates were supported by at least three ESTs (Additional file 3).

### Mapping putative pig chimeras to known human chimeric transcripts

Putative pig chimeric mRNAs were aligned to known human chimeric transcripts annotated in the chimera database (ChimeraDB 2.0) to estimate the relationship between two kinds of transcripts [17]. The fusion junctions of 21 putative pig chimeric mRNAs were matched to known human chimeric mRNAs (Additional file 4). However, only two putative pig chimera variants (AK239284 and AK349030) whose fusion junction was overlapped with that of a known human chimeric mRNA (AML1/AMP19 fusion gene) were found.

### Validation by transcriptome sequencing

We collected 396.2 million sequence reads from the transcriptome sequencing of liver tissue samples from 11 adult Bama miniature pigs (five males and six females, Additional file 5). This procedure was done to verify that the putative chimeric mRNAs were real expressed genes rather than involved in exonic coding sequences shared among multiple genes or homologous pseudogenes. The Illumina Genome Anlayzer II was employed to sequence these samples. Two length types of single-end reads, 76 and 101 nt, were generated (Additional file 5). For the uniformity of the read length, 101 nt reads were trimmed to 76 nt from a low-quality (right) end, which would increase the quality of 101 nt reads.

**Figure 2:**
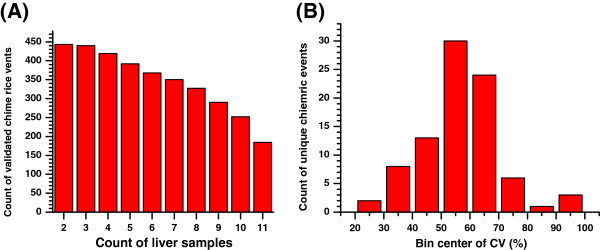
**Transcription of chimeric mRNAs in liver samples.** (**A**) Count of validated chimeric events as grouped according to the count of liver samples. A fusion junction mapped by junction reads derived from at least three different start positions or at least three samples was considered a validated chimeric event. (**B**) Distribution of unique chimeric events along the CV. The CV is the percentage ratio of the sample standard deviation to the sample mean of the junction reads for each event. The 87 unique chimeric events were put in eight bins according to the CV.

Reads from different samples were mapped on the pig genome (SGSC Sscrofa9.2/susScr2, Nov. 2009) using the Bowtie software (version 0.12.8) [18]. This Bowtie version does not report gapped alignments. Hence, a read mapped on the genome was derived from a contiguous genomic locus. In this sense, some unmapped reads may have originated from non-contiguous genomic loci and may therefore be suitable in inspecting splice junctions. Based on this fact, these unmapped reads were aligned to putative chimeric mRNA. In this step, we required that the junction reads should overlap with at least 5 nt of the sequence on either side of the chimeric junction. Furthermore, a fusion junction mapped by junction reads derived from at least three different start positions or at least three samples was considered a validated chimeric event. Consequently, up to 443 fusion junctions were validated by this strategy (Additional file 6). The 440 and 184 events were expressed in at least three and all samples, respectively (Figure 2A).

Estimation was further performed on the validity of junction reads that overlapped fusion points with a minimum of 5 nt. In the present study, reads were trimmed to 76 nt. Therefore, the length of fusion junctions was 142 nt (71 nt on either side of the fusion junction) by requiring a 5 nt overhang for read mapping fusion points. If the start position of a read located in the region from the 1^st^ to the 67^th^ nt of the fusion junction, the read was termed as a junction read. In this estimation, reads from 11 liver samples were pooled together. The 496 fusion junctions were matched by at least one read. Among these junctions, 89.3% (443/496) were overlapped by at least three reads and 89.7% (440/496) were validated by reads starting from at least three different positions (Additional file 7).

**Figure 3:**
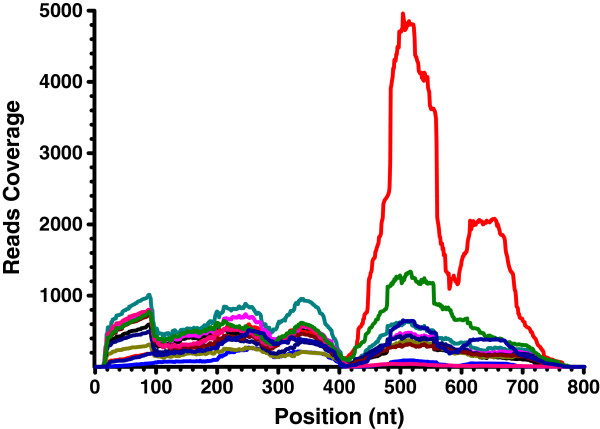
**Reads not uniformly distributed along mRNA.** The graph is an example showing that the read coverage is not uniformly distributed along the AK346347 transcript. Read coverage at a nucleotide position was determined by enumerating the total reads mapped on that position. The 11 liver samples were all represented.

Interestingly, we observed a non-uniform distribution of reads along some mRNA sequences. For example, the read coverage showed multi-peaks along the mRNA sequence of AK346347 (Figure 3). Given that chimeric mRNAs share sequences with their precursor genes, determining which reads come from chimeras is necessary. Reads mapped on the fusion junctions were derived only from chimeric mRNAs. However, reads mapped on positions away from the fusion junctions would be derived from either chimeric mRNAs or their participating parental transcripts. An apparent trough was detected in the region from 400 nt to 420 nt, corresponding to the trans-splicing site at 403 nt (Figure 3). The lower read coverage along this chimeric junction indicated the lower expression of this chimeric gene relative to its precursors. In addition, except for the trans-splicing site, other troughs were closed to the cis-splicing sites of AK346347, indicating the existence of spliced variants of AK346347 among the samples.

### Variation of expression among individuals

We used a cut-off that required junction events to be present in all samples and unique without an overlap with other chimeras to access further the differential expression of unique chimeric mRNAs without the confounding issues of tissues. This cut-off resulted in 87 unique chimeric events. The dispersion of the expression of each unique chimeric event across the samples was measured using the coefficient of variation (CV), the percentage ratio of the sample standard deviation to the sample mean of the junction reads for each event. Figure 2B represents the distribution of junction events along the CVs. The mean of the CVs was 57%, with a standard deviation of 14%, following a normal distribution (*P*>0.57, *Kolmogorov-Smirinov test*). This result implies that most of these unique chimeric events were considered middle variances in the expression.

**Figure 4:**
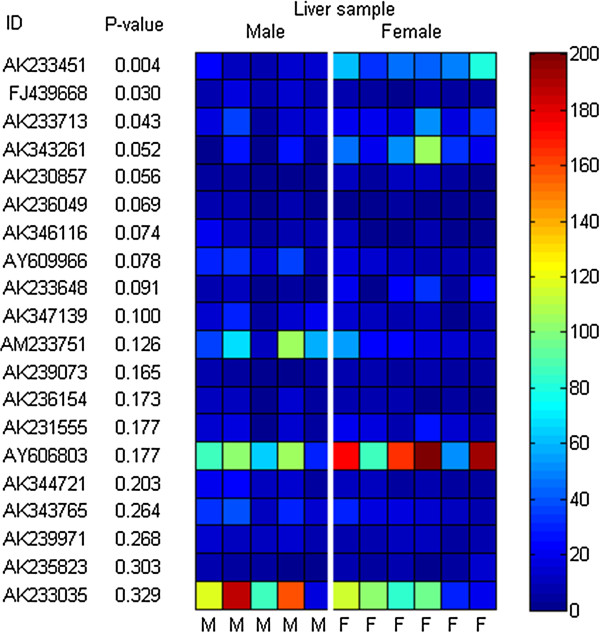
**Difference in the transcription between sexes.** The difference in the transcription of unique chimeric events between five males and six females was evaluated using a two-sided rank sum test. Top 20 events based on the *P*-value from the test were shown. The leftmost column shows the GenBank ID and the second displays the *P*-value. Each cell in the heat-map encodes the count of junction reads for each unique chimeric mRNAs in each sample. Each vertical column represents data from one sample and each horizontal row represents the relative abundance of one unique chimeric mRNAs across samples. Columns were grouped by sexes.

We compared the expression of these events between male and female samples to gain further insight. The mean CV of the females was lower than that of the males (52% versus 57%). Unique events were ordered using a nonparametric two-sided rank sum (RS) test, a statistical test that considers the difference in expression levels between male and female samples (Figure 4). The *P*-value of all events, except for three, was greater than 0.05, indicating non-significant variance in the expression of these events between sexes.

### Variation of expression among pig breeds

More attention was given to the variation in the expression of chimeric events among the pig breeds. A set of 49 nt single-end reads from three RNA-pooling samples of skeletal muscle was analysed in the same way as those from liver samples (Additional file 5, Additional file 6). These samples were obtained during embryo collection at slaughter. The first, second, and last samples were pooled using equivalent amounts of RNA from three adult female Wuzhishan, Tongcheng, and Landrace pigs, respectively. These samples may remove the difference among female individuals to some extent. The mean of the CVs was 35%, with a standard deviation of 19%, spanning 0% to 89% and following a normal distribution (*P*>0.61, *Kolmogorov-Smirinov test*).

### DNA motif in the genomic region of chimeras

To exploit the putative mechanism responsible for the generation of chimeric mRNAs, we attempted to retrieve DNA motif sequences in two non-continuous genomic loci of chimeric mRNAs using the MEME software (Motif-based sequence analysis tools, version 4.6.1) [19]. In this step, for 445 putative chimeric mRNAs, similar DNA sequences were found between the 5^′^ and 3^′^ partners (Additional file 8). Similar sequences were prevalent in the upstream, intronic, and downstream sequences, but deficient in exons (Table 1). None of the similar sequences was found in the exonic pair of the 5^′^ and 3^′^ partners. The lack of similarity in the exonic pair may result from the elimination of chimeras with long overlapping sequences in the fusion junction, implying that although we cannot entirely exclude false positive results from homologous or paralogous genes, we minimised the effect of these events on the identification as much as possible. This result agrees with the suggestion that some regulatory elements, such as transcription factor binding sites or enhancers, are highly pronounced in non-coding regions.

**Table 1:** Distribution of potential shared DNA motifs in genomic regions

**Type**	**3**^′^**Up**	**3**^′^**Exon**	**3**^′^**Intron**	**3**^′^**Down**	**Total**
5^′^ Up			72 (6)	1 (0)	46 (9)	55 (7)	174 (22)
5^′^ Exon			0 (0)	0 (0)	2 (0)	0 (0)	2 (0)
5^′^ Intron			38 (2)	0 (0)	38 (10)	42 (18)	118 (30)
5^′^ Down			66 (11)	1 (0)	43 (4)	41 (14)	151 (29)
Total			176 (19)	2 (0)	129 (23)	138 (39)	445 (81)

The table indicates the count of potential DNA motifs shared within the genomic regions of the 5^′^ and 3^′^ partners. The rows represent the genomic regions for the upstream, exon, intron, and downstream regions of the 5^′^ partner and the columns of the 3^′^ partner. Numbers in parentheses indicate the count of shared sequences that significantly match the known DNA motifs in the JASPAR CORE (*P*<0.00065 and *false motif discovery rate* < 0.05). Up: upstream; Down: downstream.

Subsequently, these shared sequences were submitted to the TOMTOM [20] software in the MEME suite (4.6.1) [19] for comparison against the database of known motifs. This database is the JASPAR CORE (version 12-Oct-2009) that contains a curated, non-redundant set of profiles derived from published collections of experimentally defined transcription factor binding sites for multi-cellular eukaryotes [21]. The 81 shared sequences significantly matched known DNA motifs in the JASPAR CORE database (*P*<0.00065 and *false motif discovery rate* < 0.05, Additional file 9). Among these matched sequences, 6 were shared in the upstream regions of both partners (*P*<0.00009 and *false motif discovery rate* < 0.042). This finding suggests that the same or similar transcription factors would bind these potential shared DNA motifs to coordinate the transcription of parental genes, which may be necessary in generating chimeric mRNAs.

The CCCTC-binding factor (CTCF) is a versatile trans-acting factor that binds distal regulatory elements such as enhancers, and CTCF binding sites are commonly distributed along the vertebrate genomes [22-26]. Thus, we placed efforts on computationally identifying potential CTCT binding sites shared in two non-continuous genomic regions of chimeric mRNAs. Four DNA motifs shared in parental genomic regions were significantly similar with known human CTCF binding sites (*P*<0.014 and *false motif discovery rate* < 0.029, Additional file 10). This result suggests that some trans-acting factors, such as the CTCT-binding factor, might bind these shared motifs to facilitate the approximation of the distal genomic parts and make up the subcellular environment for the generation of chimeric mRNAs. Communication between distal chromosomal elements would be an origin for the nuclear processes of gene fusions.

## Discussion

**Figure 5:**
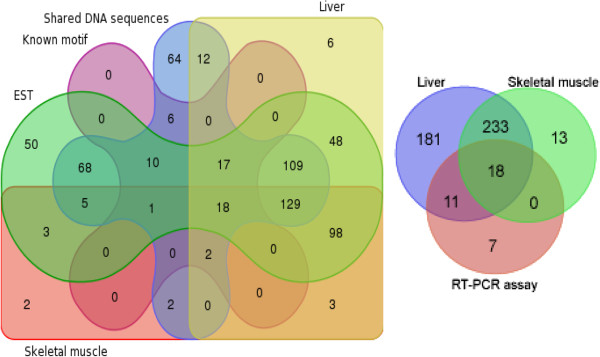
**Venn diagram showing the intersection of different groups.** The EST group represents chimeras that overlap ESTs (*n* = 557). The liver and skeletal muscle groups reveal chimeras that were present in two kind of samples (*n* = 443 and 264). The shared DNA sequences group represents chimeras with similar DNA motifs in the genomic regions of both partners (*n* = 445). The known motif group indicates the shared sequences that significant match the known DNA motifs (*n* = 81). The RT-PCR group shows chimeras validated by the RT-PCR assay (*n* = 36).

Following the hypothesis that a fusion transcription is derived by two non-continuously genomic loci, the present study revealed a list of pig chimeric mRNAs validated by the RNA-Seq and EST data (Figure 5). A set of unique chimeric mRNAs showed a middle variance among both individuals and breeds. The results provided detailed information regarding pig chimeric mRNAs and important implications for gene fusions.

Several factors including strand-switching, deep sequencing errors, or reference genome errors would result in false positive results. Therefore, we rigorously inspected each chimera using several criteria. First, all the mRNAs used in the present study have prior biological information annotated in the UCSC and NCBI databases to avoid reference genome errors as much as possible. To remove false results from homologous, paralogous, or random spurious hits, strict filtering was performed on the highly qualitative alignments of mRNAs to the *S. scrofa* chromosomes. Trans-splicing sites were then inspected for each candidate to exclude strand-switching or the random connection of two cDNAs. In addition, 14 independent samples were used to evaluate the expression of the fusion transcripts. We could not completely rule out the possibility of the creation of a false fusion in the process of cDNA library construction. However, random breakage and rejoining of two cDNAs are unlikely to happen at the exact exon boundaries of two genes and simultaneously in multiple samples. Thus, although the present identification of chimeric RNAs filters out some genuine fusion gene transcripts by stringent cut-offs, it is conservative and reliable.

**Figure 6:**
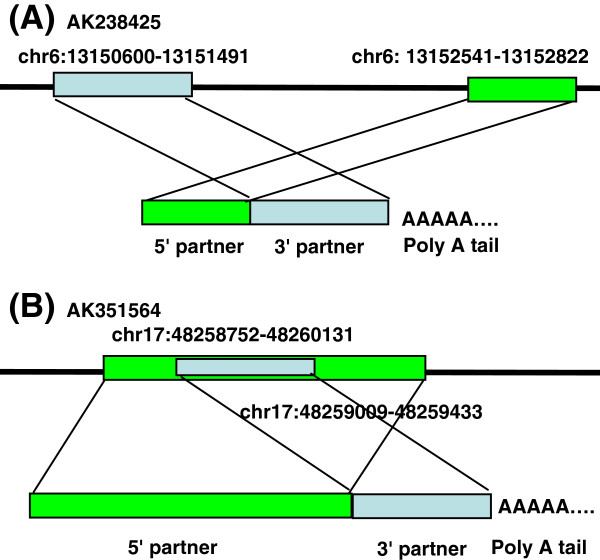
**Transcriptional read-through was infrequently involved in chimeric candidates.** The genomic and mRNA organization of AK238425 and AK351564 are depicted in the figure. (**A**) The 5^′^ partner of AK238425 is located downstream of the 3^′^ partner. (**B**) The 3^′^ partner of AK351564 lies in the body of the 5^′^ partner.

Interestingly, the transcriptional reading-through was infrequently involved in the intra-strand chimeric candidates identified in the present study. RNA-polymerase generally ends at transcriptional terminators, preventing it from reading through the downstream gene. However, in unusual cases, long transcriptions span terminators and produce new, hybrid, multi-locus transcripts [8-11]. We checked the coordinates of putative chimeras on the pig genome and found that the exons of most intra-strand chimeras were out of order compared with those found in the genome. For example, the 5^′^ partner of AK238425 is located at the downstream of the 3^′^ partner on the plus strand in chromosome 16 (Figure 6A). AK351564 is another example that a 3^′^ partner lies in the body of the 5^′^ partner (Figure 6B). Among the 150 intra-strand chimeras, 142 follow the AK238425 way, five take the AK351564 way, and two occur in the third way that the 5^′^ partner lies in the body of the 3^′^ partner. The skewed representation of chimeras in the three ways was due to the elimination of chimeras with long overlapping sequences in the fusion junction. Only one chimera follows the order that the 5^′^ partner is located in the upstream of the 3^′^ partner. However, the distance between partners is 57,234,234 nt.

During transcription in vivo, different genes frequently share the same transcription factory where nascent RNA production and RNA polymerase II seem to be localised [27,28]. For example, the *Igh* on chromosome 12 is preferentially recruited to the same transcription factory where the *Myc* gene on chromosome 15 is highly transcribed [29]. Many active genes can dynamically co-localise to shared sites of ongoing transcription, which may be induced by the classical effectors of gene expression including trans-acting factors, enhancers, chromatin modifications, and chromosomal interaction [27]. For example, CTCF can create the dynamic nature of nuclear spatial organisation of different genes by binding to the elements on distal genomic regions or different chromosomes [25,30,31]. The recruitment of different genes into shared factories is expected to have a fundamental role in gene expression, which may efficiently share limited resources or perhaps coordinate the transcription of different genes.

**Figure 7:**
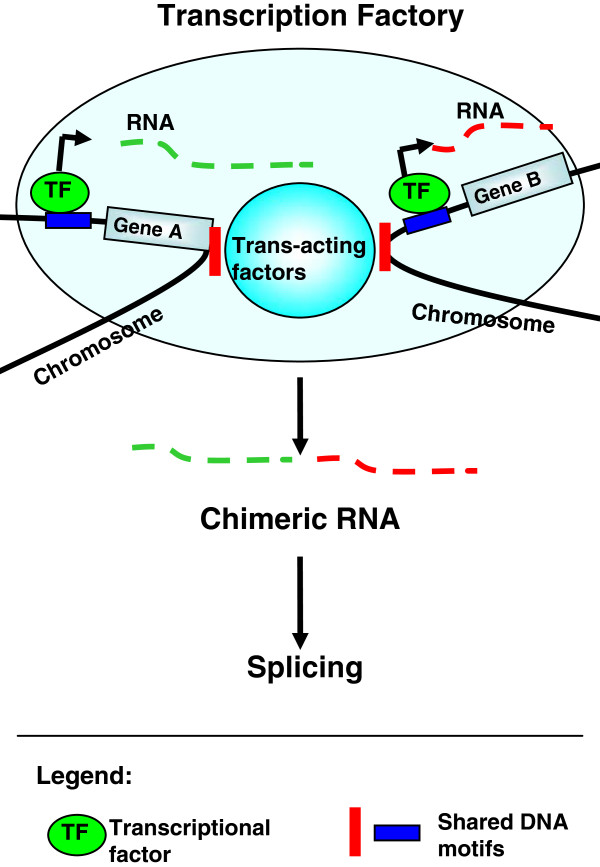
**Model for the generation of chimeric mRNAs.** Induced by trans-acting factors, such as the CTCF, parental genes dynamically co-localise to the shared transcription factory. The same or similar transcription factors coordinate the transcription of parental genes. Then, the nascent transcripts are joined together and spliced.

The co-localisation of different genes into the same transcription factories provides insights into the origin of chimeric mRNAs. We found similar sequences shared in the 5^′^ and 3^′^ partners of some chimeric mRNAs. Some shared DNA motifs significantly matched the known DNA-binding motifs. For example, four shared DNA motifs have significant similarity with known human CTCF binding sites. The CTCF can recognise and bind to different DNA motifs by its zinc-finger domains [32]. Induced by trans-acting factors, such as the CTCF, parental genes may dynamically co-localise to the shared transcription factory, and then the same or similar transcription factors coordinate the transcription of them to give birth to chimeric mRNAs (Figure 7).To some extent, this result agrees with the suggestion that short homologous sequences at the junction sites may induce the formation of chimeric mRNAs [7].

As earlier common computational methods for identifying precursor genes, a gene with the best alignments to a chimeric mRNA was considered as the precursor gene [7]. However, exons often overlap exons for some cases. For example, the 5^′^ partner of the chimeric mRNA AK343294 was precisely mapped on the exons of mRNAs AK233826, AK231250, and AK346646 in chromosome 5. Therefore, the precursor mRNA would be discretionary if multiple transcriptional start sites were present. Furthermore, the partners of chimeric mRNAs may be transcribed independently at their own transcriptional start sites that are not associated with other genes. Thus, the selection of which variant would serve as the precursor gene would need more molecular experimental identifications.

## Conclusions

The present study provided detailed information on pig chimeric mRNAs and further analysed the expression of unique chimeras among samples. Interestingly, similar DNA sequences widely shared in the two non-continuously genomic regions of chimeric mRNAs. Similar DNA sequences that share in the upstream regions of both partners significantly matched the known transcription factor binding sites in the JASPAR CORE database, suggesting the potential coordinated transcription of the parental genes. In addition, possible CTCF binding sites were also observed in the parental genomic regions. We supposed that trans-acting factors, such as CTCF, would induce the spatial organisation of parental genes to the same transcriptional factory so that parental genes would be coordinatively transcribed to give birth to chimeric mRNAs. Although this hypothesis needs further experimental evidence, it will provide useful information for the investigation of the mechanism for the generation of chimeric mRNAs. Overall, our results will aid in the further understanding of chimeric mRNAs.

## Methods

### Chimeric mRNA identification

The BED format table of all pig mRNAs were analysed for further study using the Galaxy [33-35] in the UCSC Table Browser (February 2011). According to the annotation of that table, GenBank pig mRNAs were aligned against the pig genome (SGSC Sscrofa9.2/susScr2, Nov. 2009) using the Blat program [36]. The alignment with the highest base identity was found when a single mRNA was aligned in multiple places. Only alignments with a base identity level within 0.5% of the best and at least 96% base identity with the genomic sequence were kept ( http://genome.ucsc.edu/). An entry in that BED table annotates a chromosomal locus of an mRNA. We extracted mRNAs aligned to two non-contiguous loci. We required alignments from non-contiguous loci without long similar sequences at the putative junction sites to remove homologous, paralogous, or random spurious hits. In accommodating small errors in alignment that occur at the edges of the alignment, we only allowed overlaps or gaps of up to 10 nt within the fusion junction. Using the Circos software [37], we represented the genome-wide distribution of putative chimeric mRNAs in Figure 1.

To validate putative chimeras by external experimental evidence, we aligned predicted chimeras to the EST sequences downloaded from the UCSC (May 2012) using the BLAST program (Basic Local Alignment Search Tool, version 2.2.26+) [38-40] with default parameters except at least 96% base identity. The candidate was retained for analysis when at least 20 nt of the sequence on either side of a putative fusion point overlapped ESTs. To compare with known human chimeras, we aligned pig putative chimeras to human chimeric mRNAs downloaded from the ChimeraDB 2.0 [17] using the BLAST with default parameters (May 2012).

### Inspection of splice sites

As previously described [41], we prepared an artificially fused genomic DNA sequence for putative chimeras by joining the genomic sequences of the 5^′^ and 3^′^ partners. The fusion transcript candidate was then aligned to the corresponding artificially fused genomic sequence using the SIM4 program (version 2002-03-03) [16] with default parameters. The alignment around the fusion point was inspected to take into account consensus splice signals.

### Validation by RT-PCR

We obtained total RNAs from Tongcheng pig tissues (liver, kidney, spleen, heart, lung, testis, ovary, embryo, skeletal muscle, small and large intestine) using the RNA Extraction Kit (BioTeke). The cDNA was prepared by reverse transcription using the Strand cDNA Synthesis Kit (BioTeke) with random hexamer priming and oligo dT’s. PCR products covering the junction position were amplified using primers designed according to the hybrid transcripts (Additional file 2). PCR amplification was performed using the following thermocycling protocol: initial denaturation at 95°C for 4 min, followed by 30 cycles of denaturation at 95°C for 30 s, annealing at 60°C for 30 s, and elongation at 72°C for 30 s. The PCR products were then analyzed, cloned, and sequenced.

### Validation by RNA-seq data

Up to 400 million sequence reads from deep sequencing the transcriptome of pigs were recently acquired in our lab. In brief, the following steps were used for transcriptome sequencing using the Illumina Genome Analyser II at Shanghai Biotechnology Co., Ltd. We isolated mRNA from 10 μg of total RNA with an RNA integrity number (RIN) ≥ 8. The isolated mRNA was then fragmented and converted into double-stranded cDNA. The ends of cDNA were ligated to adapters. The fragments with 200 to 300 base pairs in length were amplified by PCR to make a library. Finally, the library was sequenced to yield single-end reads.

A set of reads was derived from the transcriptome of the liver tissue samples obtained from 11 adult Bama miniature pigs (five males and six females, Additional file 5). Reads with a Phred quality score lower than 20 were filtered out. The length of the reads from eight pigs was 76 nt, whereas that from the other three pigs was 101 nt. To obtain uniform lengths of reads, the 101 nt were trimmed from the low-quality (right) end of each read to only 76 nt before mapping. The remaining reads were aligned to the pig genome (SGSC Sscrofa9.2/susScr2, Nov. 2009) using the Bowtie software (version 0.12.8) [18] with default parameters except maximum two mismatches, unique mapping, and trimming from 101 to 76 nt for the three samples.

The present version of the Bowtie program (version 0.12.8) does not report gapped alignments. Thus, a read mapped on the genome was derived from a contiguous locus in the genome. However, some unmapped reads may arise from non-contiguous genomic loci, making them suitable for inspecting splice junctions. The unmapped reads were further aligned to the putative chimeric mRNAs by the Bowtie program with default parameters except maximum two mismatches and trimming from 101 to 76 nt for the three samples. The previously unmapped reads that were matched on the putative junctions with an overlap of at least 5 nt on either side of the RNA junction were remained for further analysis.

Another set of 49 nt single-end reads from three equivalently pooled RNA samples of skeletal muscle was analyzed as described above (Additional file 5). These samples were extracted during embryo collection at slaughter. The first, second, and last samples were pooled using equivalent amounts of RNA from three adult female Wuzhishan, Tongcheng, and Landrace pigs, respectively.

CV was calculated to represent the variance in the expression. The reads uniquely mapped on the pig genome and the junction reads were pooled together to reveal the read coverage along the transcript. The RS test was used to evaluate the difference in the expression levels between the male and female samples.

### DNA motif identification

The MEME software (version 4.6.1) [19] with default parameters (except DNA alphabet, zero or one occurrence of each motif per sequence, motif width between 10 and 30 nt, and maximum one motif to find) were used to search similar DNA sequences within two non-continuous genomic sequences of chimeric mRNAs. Then, using the TOMTOM [20] tool, similar DNA sequences were compared with the database of 476 known motifs, the JASPAR CORE (version 12-Oct-2009).

## Competing interests

The authors declare that they have no competing interests.

## Authors’ contributions

LM designed, analysed, interpreted, and drafted the manuscript. SY and ZT handled the RNA sequencing. WZ and TZ performed the molecular genetic studies. KL conceived the study and participated in its design and coordination. All authors have read and approved the final manuscript.

## Supplementary Material

Additional file 1 **Putative chimeric mRNAs.** The file lists the information on the putative chimeric mRNAs, including GenBank identifier, fusion type, overlap or gap between partners, junction site in the transcript, trans-splicing signals, and annotation in GenBank, as well as the chromosome location of the partners. Click here for file

Additional file 2 **Putative chimeric mRNAs validated by RT-PCR.** The file shows the results of the RT-PCR assay. Click here for file

Additional file 3 **Putative chimeric mRNAs validated by ESTs.** The table lists the chimeric mRNAs validated by ESTs. The columns of the table consist of the GenBank ID of putative chimeric mRNAs, the number of ESTs that overlap at least 20 nt of the sequence on either side of a putative fusion point, as well as the GenBank ID of each EST. Click here for file

Additional file 4 **Pig putative chimeric mRNAs similar to known human chimeric mRNAs.** Pig putative chimeric mRNAs were aligned to known human chimeric transcripts. The table represents the BLAST results and the annotations of human chimeric mRNAs in ChimeraDB version 2.0. Click here for file

Additional file 5 **Information on RNA-seq reads.** The raw reads, the cleaned reads, the uniquely mapped reads on the genome, the multi-mapped reads on the genome, the un-mapped reads on the genome, and the junction reads are shown in the table. Click here for file

Additional file 6 **Pig chimeric mRNAs validated by RNA-seq reads.** The table shows the pig chimeric mRNAs validated by the RNA-seq reads derived from liver tissue and skeletal muscle. A fusion junction mapped by junction reads derived from at least three different start positions or at least three samples was considered a validated chimeric event. The junction reads should overlap with at least 5 nt of the sequence on either side of the chimeric junction. Click here for file

Additional file 7 **Evaluation on the junction reads.** Figure (A) shows the count of the fusion junctions based on the count of junction reads. Figure (B) represents the count of the fusion junctions based on the count of positions that junction reads start. Click here for file

Additional file 8 **Putative DNA motifs shared in the genomic regions of the partners.** The table gives some information on the putative DNA motifs shared in the genomic regions of the partners. Click here for file

Additional file 9 **Similar DNA sequences that match known DNA motifs in the JASPAR CORE database.** The table shows similar DNA sequences matched known DNA motifs in the JASPAR CORE database. Click here for file

Additional file 10 **Potential CTCF binding motifs.** The table shows potential CTCF binding motifs. Click here for file
