# Supplementary material for: Identification and analysis of pig chimeric mRNAs using RNA sequencing data
Source: BMC Genomics. 2012 Aug 28;13:429. doi: 10.1186/1471-2164-13-429 (PMC3531304; doi:10.1186/1471-2164-13-429)
Supplement: Additional file 2 — Putative chimeric mRNAs validated by RT-PCR. The file shows the results of the RT-PCR assay. [file 1471-2164-13-429-S2.pdf]

1. RT-PCR results for verification of identified chimeric mRNA

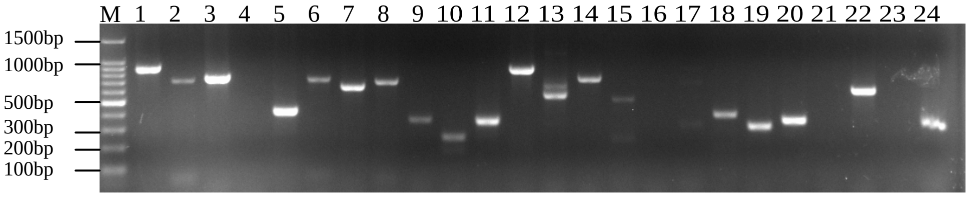

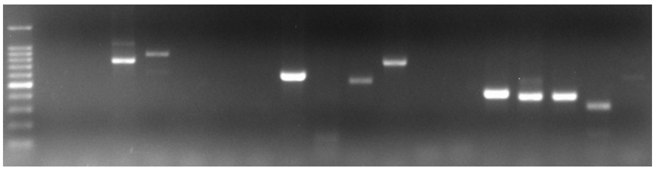

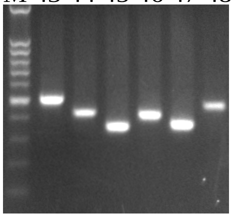

M: Maker ladder (Top-down: 1500, 1000, 900, 800, 700, 600, 500, 400, 300, 200 and 100bp)

2. Design for PCR

No.	ID	Size	Forward prime (5'-3')	Reverse prime (5'-3')	Validation
1	AB044390	885	CGAGTTCTCGAGGGTCAGTC	ATTGCTGGTTGGGTCTTCAC	+
2	AF202775	714	CCTCTTCCTGGCACACTCTC	CTGGCTCAATGGCTTCTAGG	+
3	AK230733	739	GCTCATCACACCTGCAGAAA	GACGGAACATCCAGTGTCCT	+
4	AK231555	466	TTGAAGCGGATAGTCGGAAG	GCGCCATCAAAGATGAAAAT	—
5	AK232284	409	TCCAAACACCAAACAGTGGA	AAGACGACGCTGGTTTCTGT	+
6	AK233462	722	CCCTCCCTCTCTGAGCTTGA	TGCTTGTTCTGCACCTCGAT	+
7	AK234693	633	GACAGGTTCTGGACGGTGAT	ATCACACAGGCGGTAGCTCT	+
8	AK234805	704	TCTGCGTGCTGTATCCTGAC	GATTTGGTCAGCCAACCAGT	+
9	AK234852	347	TCTCCTGAGGCTGTTGCTGTAG	GCTACTTGTGCTCCTGGAAGGT	+
10	AK235089	245	AGATCAGGCTTGGGTTGATG	TCCTGTTCTCTGGGGTTTTG	+
11	AK236869	346	GAGGACCTCAAAGTGGGACA	CAATTAGGCTCCCAATTCCA	+
12	AK236878	887	CAGACACTGGTGTGGGAATG	GGGGAGATCATCTGAGTCCA	+

13	AK236900	648	CATCAGCATCTGGGGTTTCA	CCCACAGTGGCCAGGATAAT	+
14	AK237523	732	TCATGGTCCTGGAGATCGTG	TGGTTGGTGACCCTGTTGAG	+
15	AK238006	511	TGGTGTGCTGTGAATTGGTT	TGGTTCAATACGATCCGACA	+
16	AK239166	512	CTGAGGAATCGTCCTCCGTA	TTTTTGGGTGCCTTTTTGAC	—
17	AK344029	308	CGCCACCAACTCTGAGACTG	GTGTCTCCAGGAGGGACTGG	weak
18	AK344580	385	ATGCTGCAGAGGAGGGTCTA	GGAGGTTAGGGCATGTGAAA	+
19	AK345292	301	GGACCCAACAAGGTTGAGAA	TGGCCATCTCTTTCTTGTCC	+
20	AK345536	336	TGACGAGAGCAAGAAGCAGA	ATCAGAGACCCCTCCAGGTT	+
21	AK345923	537	CGAAATGAAAGGCTCACCTC	GGCTCAGGAGCTTGAAGTTG	—
22	AK346264	590	CCTGTGGTGGTCATTGACAG	CCTTCTGGGACAGTGTGGAT	+
23	AK346334	379	CCCAGAGCACAGAGTTCCTC	CATCTTTGCTGCTGGAATCA	—
24	AK346631	303	TCGAGCCTCAGGTAACAGCA	TGAAGGGTACGTCGCTCAGA	+
25	AK348034	533	CCCGTCTTCTCCCAGTTGTA	GAGCCTTTCCAGCAAATGAG	—
26	AK348198	651	CTATCCATTTGGGACCATGC	CTGGTCCACACCAGGTTCTT	—
27	AK348615	787	CACAGAGACGCCTCACGAAC	TTGGCAATAGCCGACTTCCT	+
28	AK349538	884	CCCAGTGTCCCAATCTGACT	TCCTTTTCTCCCCTGAGGAT	+
29	AK349871	606	GGGACTCTCTGGAGAACACG	GCTACCAGACAGGTCGAAGC	—
30	AK350634	621	CTGCCTGGAGAACCTGAAAG	GGATGAATGGCTTCTGCATT	—
31	AK351075	402	TTGAAGGGCTGTTTGGACCT	CACTGGTGCTCTGGACCTTG	—
32	AK352450	573	GCTTCCTCCAGTTGCTGATG	ATCCTGATCCCACACGTCTC	+
33	AK352469	587	GTTTCTCGAGCCACCAAACC	CAGCAGGAGGGAGAGAGGAA	—
34	AY039112	531	GGCTGGTCTGCTTGAGAAAC	TTGCCAATTCCCAGCTTATC	+
35	AY639873	751	CCAAAAGCGGTATAGCTTGC	ATAGTCACTCTGGGGCATGG	+
36	AY705921	279	GAGCCCCTGCAATTTGGTAG	CAGCAAAGAGCGAGGTGACA	—
37	DQ333199	788	CAGTCCCTCCAGAAAATGGA	CATGGTTCTTGTGGTCGTTG	—
38	DQ673096	395	TGTTCTCAAACCTGGCATGG	CAATGGCAGCATCACCAGAT	+
39	EF576923	380	AGGAACCGACAGGTCACTTTTA	GAGAAGTTTTGAGGTGGCAAAC	+
40	EF619344	370	GCACCTGGAAGCTAGTGGAC	TCGTAAGTGCGAGTGCAAAC	+
41	FJ200454	290	GGACGACTCCAAGACATGGT	GTGCTCACTGGTGAAGATGG	+

42	S82664	530	ACGTGCAGGCCAGTAAGTCT	TACCTTCCGGATCAGCAAAC	weak
43	AK232092	498	ATCAACTGCTCCGAAGGCTA	TGGAGAGCAGCAAACACTTG	+
44	AK232429	426	CCGAAATGGAAAACTGGGTA	AACCTGCAACCTCATGGTTC	+
45	AK233435	350	GCCTTCGACACCAATATCGT	TCGAGGGGATCAAAACAGAC	+
46	AK346226	409	CCGAGAGGCCATTCTGATAG	CCTTTCATGAACCGAGTGGT	+
47	AK351319	365	GTCTGTCATCTCCCGCCTTA	CCTCACAGCTGAGGGTAAGC	+
48	AK231608	474	TTGCAACAAGAAGCATCCAG	TTTCAGTGGTTGGTGGAACA	+
