# Supplementary material for: Identification and analysis of pig chimeric mRNAs using RNA sequencing data
Source: BMC Genomics. 2012 Aug 28;13:429. doi: 10.1186/1471-2164-13-429 (PMC3531304; doi:10.1186/1471-2164-13-429)
Supplement: Additional file 5 — Information on RNA-seq reads. The raw reads, the cleaned reads, the uniquely mapped reads on the genome, the multi-mapped reads on the genome, the un-mapped reads on the genome, and the junction reads are shown in the table. [file 1471-2164-13-429-S5.doc]

**Table 1 Information on RNA-seq reads from liver samples**

| Sample No. | Sex | Reads length (nt) ## | Raw reads (million) | Cleaned reads (>= Q20 value) (million) # | Unique mapped reads on genome (million) | Multi mapped reads on genome (million) | Non-mapped reads on genome (million) | Count of fusion junction reads |
| --- | --- | --- | --- | --- | --- | --- | --- | --- |
| 1 | male | 76 | 31.7 | 30.0 | 15.5 | 3.0 | 11.5 | 8366 |
| 2 | male | 76 | 35.2 | 33.3 | 16.0 | 3.2 | 14.1 | 10765 |
| 3 | female | 76 | 36.4 | 34.5 | 15.4 | 3.3 | 15.9 | 8743 |
| 4 | male | 76 | 34.8 | 32.8 | 7.6 | 1.7 | 23.5 | 3840 |
| 5 | female | 76 | 34.8 | 32.7 | 7.5 | 1.7 | 23.5 | 5756 |
| 6 | female | 76 | 35.4 | 32.7 | 10.5 | 2.3 | 19.9 | 6526 |
| 7 | male | 76 | 30.9 | 28.1 | 11.6 | 2.5 | 13.9 | 7626 |
| 8 | female | 76 | 32.0 | 29.0 | 11.2 | 2.4 | 15.4 | 8255 |
| 9 | female | 101 | 33.2 | 28.9 | 12.2 | 2.1 | 14.6 | 2991 |
| 10 | female | 101 | 33.4 | 28.0 | 12.6 | 2.4 | 13.0 | 4751 |
| 11 | male | 101 | 35.4 | 31.1 | 11.3 | 1.9 | 17.9 | 2854 |

# Reads with a Phred quality score lower than 20 were filtered out.

## The 101 nt were trimmed from the low-quality (right) end of each read to only 76 nt before mapping.

**Table 2 Information on RNA-seq reads from RNA-pooling samples of skeletal muscle**

| Sample No. | Breeds | Reads length (nt) | Raw reads (million) | Cleaned reads (>= Q20 value) (million) # | Unique mapped reads on genome (million) | Multi mapped reads on genome (million) | Non-mapped reads on genome (million) | Count of fusion junction reads |
| --- | --- | --- | --- | --- | --- | --- | --- | --- |
| 1 | Wuzhishan | 49 | 11.9 | 11.7 | 6.4 | 2.1 | 3.1 | 4443 |
| 2 | Tongcheng | 49 | 12.6 | 12.2 | 6.7 | 2.1 | 3.4 | 4840 |
| 3 | Landrace | 49 | 12.6 | 12.3 | 6.9 | 2.1 | 3.3 | 5712 |

# Reads with a Phred quality score lower than 20 were filtered out.
