# Supplementary figures and images for: Identification and analysis of pig chimeric mRNAs using RNA sequencing data
Source: BMC Genomics. 2012 Aug 28;13:429. doi: 10.1186/1471-2164-13-429 (PMC3531304; doi:10.1186/1471-2164-13-429)

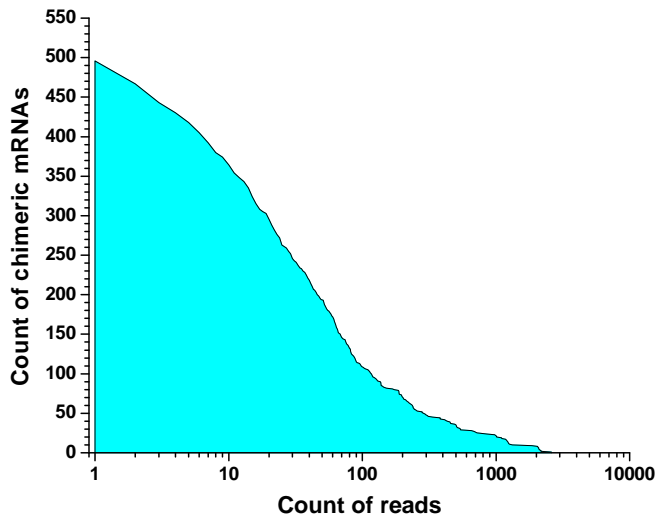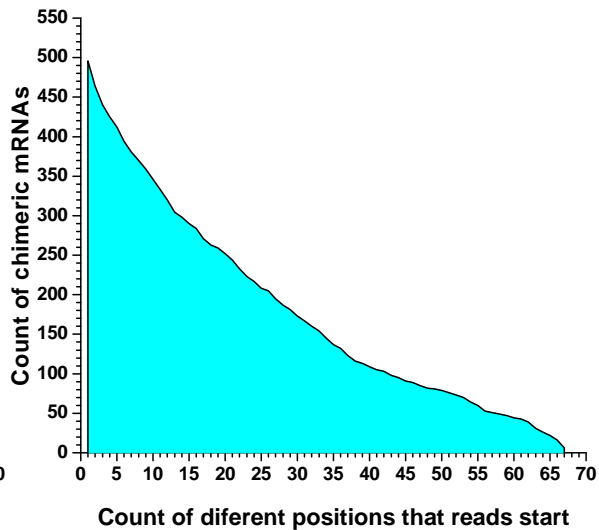

Supplement: Additional file 7 — Evaluation on the junction reads. Figure (A) shows the count of the fusion junctions based on the count of junction reads. Figure (B) represents the count of the fusion junctions based on the count of positions that junction reads start. [file 1471-2164-13-429-S7.pdf]
